# Antisense Oligonucleotide Against Clusterin Regulates Human Hepatocellular Carcinoma Invasion Through Transcriptional Regulation of Matrix Metalloproteinase-2 and E-Cadherin

**DOI:** 10.3390/ijms130810594

**Published:** 2012-08-23

**Authors:** Dong Chen, Yan Wang, Kejun Zhang, Xuelong Jiao, Bomin Yan, Jun Liang

**Affiliations:** 1 Department of General Surgery, the Affiliated Hospital of Qingdao Medical College, Qingdao University, Qingdao 266003, China; E-Mails: chendong.sdqd@yahoo.com.cn (D.C.); wlsdermyy@163.com (K.Z.); Jiaoxuelong@163.com (X.J.); 2 Department of Endodontics, School of Stomatology, Shandong University, Jinan 250012, China; E-Mail:wangyan1965@sdu.edu.cn; 3 Department of Oncology, the Affiliated Hospital of medical college, QingDao University, QingDao 266003, China; E-Mail: Yanbm818@yahoo.com.cn

**Keywords:** hepatocellular carcinoma, metastasis, clusterin

## Abstract

Secreted clusterin (sCLU) has been shown to be overexpressed in metastatic hepatocellular carcinoma (HCC) tissue, and its overexpression in HCC cells increases cell migration and the formation of liver metastatic tumor nodules *in vivo*. In this study, we tested the hypothesis that sCLU plays a role in the invasiveness of human HCC and may be associated with its metastatic spread. HCCLM3, a human hepatocellular carcinoma cell line, was transiently transfected with an antisense oligonucleotide (ASO) against sCLU (OGX-011). HepG2 liver hepatocellular cells were transiently transfected with the pc.DNA3.1-sCLU plasmid to overexpress sCLU, and subsequently evaluated for effects on invasion and the expression of molecules involved in invasion. We observed that suppression of the *sCLU* gene significantly reduced the invasive capability of the highly invasive HCCLM3 cells, and *vice versa* in the low invasive HepG2 cell line. The results revealed that knockdown of *sCLU* by *OGX-011* resulted in a significant increase in the expression of E-cadherin and a decrease in *matrix metalloproteinase-2* (*MMP-2*) gene transcription. Overexpression of sCLU by transfection with pc.DNA3.1-sCLU significantly decreased the expression of E-cadherin and increased *MMP-2* gene transcription. These data were further verified by reverse transcription-PCR and Western blot analysis. A significant reduction in MMP-2 expression and an increase in E-cadherin expression in sCLU-knockdown HCCLM3 cells were observed, as well as a significant increase in MMP-2 expression and a decrease in E-cadherin expression in HepG2 cells overexpressing sCLU. These data indicate a role for sCLU in augmenting MMP-2 transcription and decreasing E-cadherin expression. Our data show the involvement of sCLU in human HCC invasion, and demonstrate that silencing *sCLU* gene expression inhibits the invasion of human HCC cells by inhibiting MMP-2 expression and promoting E-cadherin expression. Thus, OGX-011 could be an effective therapeutic agent for HCC.

## 1. Introduction

Hepatocellular carcinoma (HCC) is one of the most common and aggressive tumors. The prognosis of HCC is extremely poor with a five-year survival rate of less than 5% for late primary HCC [1]. Despite recent advances in the diagnosis and treatment of HCC, the mortality rate of HCC remains high [2,3]. The poor prognosis of HCC patients is mainly attributed to the high rate of intrahepatic metastasis after treatment [4].

Epithelial-mesenchymal transition (EMT) is a key event in the tumor invasion process whereby epithelial cell layers lose polarity and cell-cell contacts and undergo a dramatic remodeling of the cytoskeleton [5]. A hallmark of EMT is the loss of E-cadherin expression [5]. E-cadherin is a central component of cell-cell adhesion junctions in the maintenance of the cell polarity and environment [6,7]. In HCC, loss of E-cadherin expression is associated with tumor invasiveness, metastasis, and prognosis [6] Matrix metalloproteinases-2 (MMP-2) are also involved in EMT in HCC [8,9]. An increase of MMP-2 expression is associated with tumor invasiveness, metastasis, and prognosis [10]. However, the regulatory mechanism of E-cadherin and MMP-2 is controversial and consensus has not been reached.

Clusterin (CLU) is a ubiquitously expressed glycoprotein that has been implicated in a variety of physiological processes, including cell–cell interactions, lipid transport, tissue remodeling, chaperone activity, and apoptosis [11,12]. It was first described as a protein isolated from ram testis fluid with the ability to cluster sertoli cells [13]. A growing body of evidence suggests the prominent role of CLU upregulation in tumor pathogenesis and progression. Upregulated CLU expression has been reported in cancers of the breast [14], ovary [15], colon [16,17], prostate [18], kidney [19], gastric organs [20] and HCC [21,22]. Interestingly, high levels of secreted CLU (sCLU) expression have been associated with migration, invasion, and metastasis, including HCC [19,21,23].

It has been shown that clusterin is overexpressed in metastatic human HCC tissue compared to nonmetastatic HCC tissue [21], implicating a role for clusterin in HCC progression. A recent study showed that the down-regulation of clusterin sensitizes cells to chemotherapy and radiotherapy and decreases their metastatic potential [24,25]. Although CLU expression has been associated with various human malignancies, the mechanisms by which CLU promotes cancer progression and metastasis have not been elucidated. Chou *et al*. [26] found that CLU regulates epithelial-to-mesenchymal transitions (EMT) and aggressive behavior of human lung adenocarcinoma cells through modulating ERK signaling and Slug/E-cadherin expression.

In hepatocellular carcinoma, it is not clear whether clusterin silencing inhibits the invasion and metastasis of this disease. In the present study, we provide evidence that clusterin plays an important role in HCC invasiveness by increasing MMP-2 expression and decreasing E-cadherin expression.

## 2. Results and Discussion

### 2.1. Clusterin Expression in Human HCC Cells

**Figure 1:**
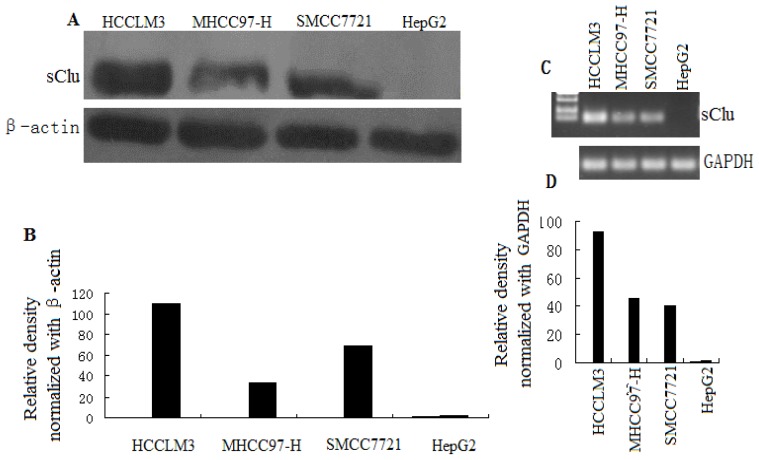
sCLU expression in HCC cells. (**A**) Whole cell lysates were prepared, and Western blot analysis was done to evaluate sCLU protein levels in SMCC7721, HepG2, HCCLM3, and MHCC97-H cells. Blots were reprobed with β-actin antibody to verify equal loading of proteins; (**B**) Histogram represents the density of bands in A normalized with β-actin; (**C**) Representative PCR of reverse-transcribed total RNA extracted from SMCC7721, HepG2, HCCLM3, and MHCC97-H cells. To normalize CLU gene expression, GAPDH cDNA was also amplified in the same samples; (**D**) Histogram represents density of bands in B normalized with GAPDH.

Clusterin has been implicated in many malignancies, but its role in HCC metastasis is not well defined. Therefore, we examined the expression of clusterin in different HCC cell lines. Western blot analysis and RT-PCR showed a significantly higher level of sCLU expression in cells with a high metastatic capacity (HCCLM3, MHCC97-H) and low sCLU expression in cells with low metastatic capacity (SMCC7721). Moreover, no sCLU expression was found in HepG2 cells (Figure 1). Therefore, it appears that sCLU expression is associated with the metastatic capacity of HCC cells. In the present study, HCCLM3 and HepG2 cells were chosen for further investigation.

### 2.2. *sCLU* Knockdown Decreases Cell Invasion

**Figure 2:**
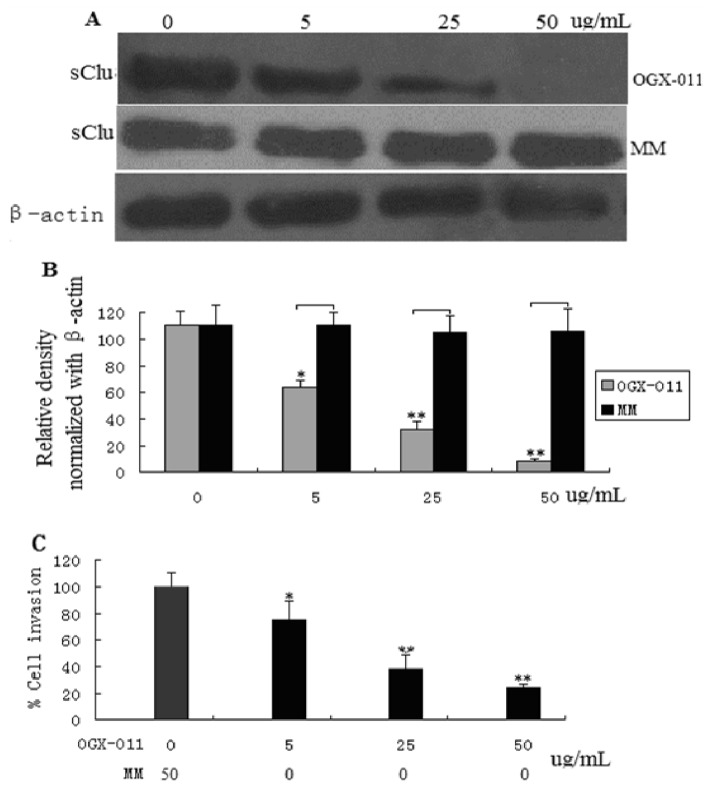
Effect of *sCLU* knockdown on the invasive behavior of HCCLM3 cells. (**A**) HCCLM3 cells were treated with 5.25, and 50 μg/mL antisense oligonucleotide (ASO) against sCLU (OGX-011) or MM (mock control) for 48 h. sCLU expression was detected by Western blotting; (**B**) Histogram represents the density of bands in A normalized with β-actin; (**C**) Effect of *sCLU* knockdown on cells subjected to invasion assays using a two-chambered invasion apparatus. The histogram shows percent inhibition of HCCLM3 cell invasion. The experiment was done in triplicate and the value obtained from MM-treated cells was set at 100%. Each bar represents mean ± SE (*n* = 3); * *p* < 0.05, ** *p* < 0.01.

To address the role of sCLU in HCC invasion, knockdown of *sCLU* was achieved by transfecting HCCLM3 cells with antisense oligonucleotide (ASO) against sCLU (OGX-011), followed by an evaluation of sCLU expression (Figure 2A,B). Since we hypothesized that sCLU is involved in HCC invasion, and due to the fact that HCCLM3 cells represent advanced metastatic cancer, we selected this particular cell line for our studies. OGX-011 dose-dependently decreased sCLU expression with maximum effect observed at a concentration of 50 μm/L 48 h post-transfection (Figure 2A,B). To further show the effects of *sCLU* knockdown, HCCLM3 cells were subjected to invasion assays. *sCLU* knockdown in HCCLM3 cells caused an 80% decrease in cell invasion (Figure 2C), demonstrating the essential role of sCLU in conferring invasive properties to HCCLM3 cells.

### 2.3. *sCLU* Overexpression Increases Cell Invasion

**Figure 3:**
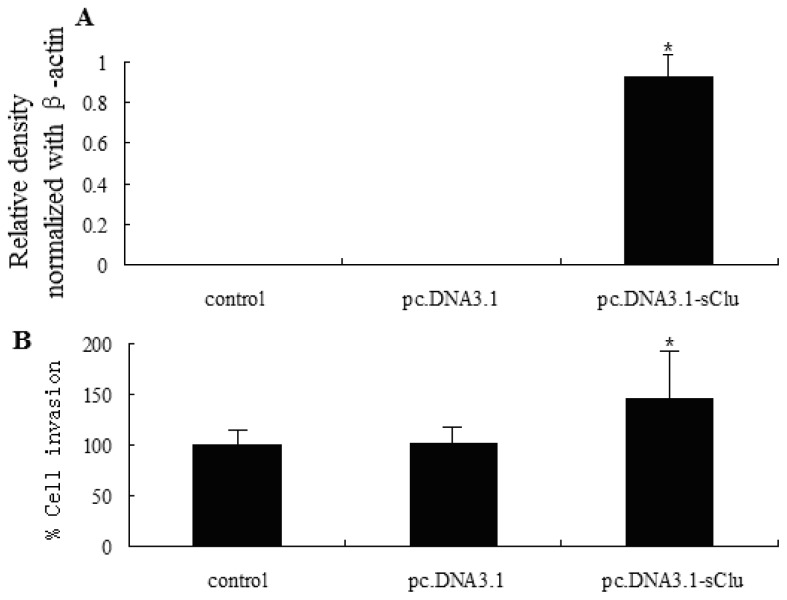
Effect of sCLU overexpression on the invasive capability of HepG2 cells. (**A**) Western blot analysis of sCLU expression in cells transfected with pc.DNA3.1 (vector) or pc.DNA3.1-sCLU. Histogram represents the relative density of sCLU bands normalized to β-actin; (**B**) Histogram showing the invasive capability of transfected HepG2 cells. The experiment was done in triplicate and the value obtained from pc.DNA3.1 transfected cells was set at 100%. Each bar represents mean ± SE (*n* = 3); * *p* < 0.05.

HepG2 cells transfected with the pc.DNA3.1-sCLU plasmid displayed a significant increase in *sCLU* expression levels compared to vector control. Overexpression of *sCLU* was confirmed by Western blot analysis 36 h post-transfection (Figure 3A). Since *sCLU* expression levels were very high 36 h after transfection (data not shown), we selected this time point for further studies. We analyzed the effect of *sCLU* overexpression on the invasive capability of HepG2 cells. As shown in Figure 3B, overexpression of HepG2 significantly increased (*p* < 0.05) the number of invasive cells. These data further support our hypothesis that *sCLU* confers invasive characteristics to cells during human HCC development.

### 2.4. Effect of *sCLU* Gene Knockdown on *MMP-2* Expression

**Figure 4:**
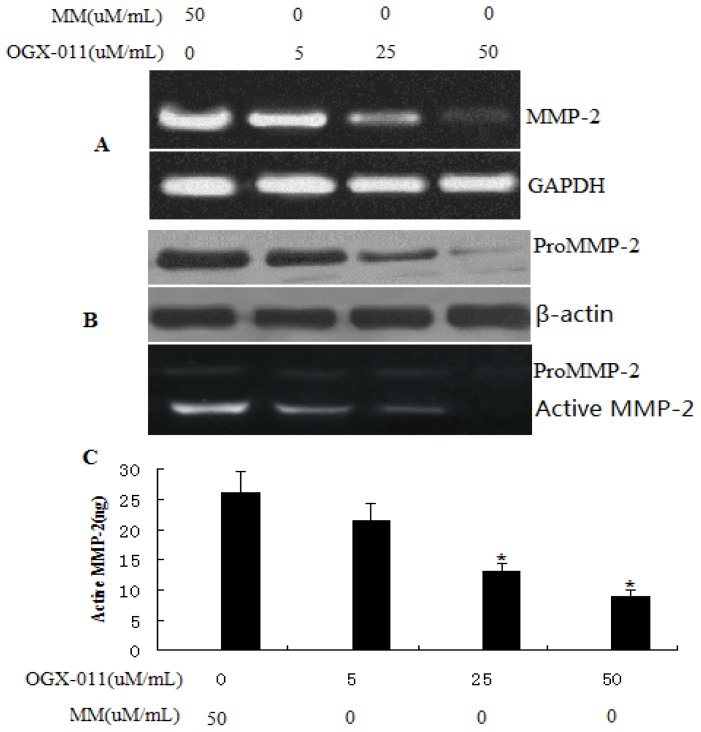
Effect of *sCLU* knockdown in HCCLM3 cells on *MMP-2* gene expression. (**A**) Representative images showing the expression of *MMP-2* mRNA, as determined by RT-PCR; (**B**) Western blot analysis to evaluate pro-MMP-2 protein expression in *sCLU*-knockdown HCCLM3 cells. Blot was reprobed with β-actin antibody to verify the equal loading of proteins; (**C**) Gelatin zymogram showing activity of pro-MMP-2 and active *MMP-2* in OGX-011 and MM treated HCCLM3 cells. Columns, mean of quadruple experiments; bars, SD. * *p* < 0.05.

Increased MMP activity is considered important for the increased capability of cancerous cells to traverse the membrane and invade and metastasize to distant sites [27,28]. We analyzed the effect of *sCLU* gene suppression on the expression and activity of *MMP-2*. HCCLM3 cells transfected with OGX-011 exhibited a significant reduction in the expression of *MMP-2* mRNA (Figure 4A) and in pro-MMP-2 protein levels (Figure 4B). Gelatin zymography was done to assess MMP-2 activity in cultured medium from *sCLU* knockdown cells, and we observed a significant decrease in MMP-2 activity (Figure 4C).

### 2.5. Effect of *sCLU* over Expression on *MMP-2* Expression

**Figure 5:**
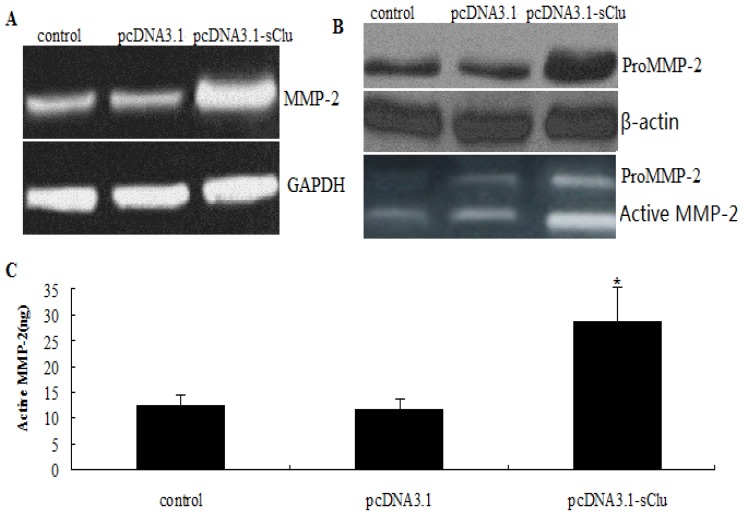
Effect of *sCLU* overexpression on *MMP-2* expression and activity. (**A**) HepG2 cells were transfected with pc.DNA3.1-sCLU or pcDNA3.1 for 36 h. Representative images showing expression of *MMP-2* mRNA in transfected cells, as determined by RT-PCR; (**B**) Western blot analysis to evaluate pro-MMP-2 protein expression in pc.DNA3.1-sCLU or pc.DNA3.1-transfected HepG2 cells. Blot was reprobed with β-actin antibody to verify the equal loading of proteins; (**C**) Gelatin zymogram showing activity of pro-MMP-2 and active *MMP-2* in pc.DNA3.1-sCLU or pc.DNA3.1-transfected HepG2 cells. Columns, mean of quadruple experiments; bars, SD. * *p* < 0.05.

We have shown that *sCLU* gene knockdown-mediated decreases in *MMP-2* mRNA levels result in decreased levels of MMP-2 protein, and hence decreased MMP-2 activity. Next, we determined the effect of *sCLU* overexpression on the expression and activity of MMP-2. HepG2 cells transfected with the pc.DNA3.1-sCLU construct exhibited a significant increase in *MMP-2* mRNA levels (Figure 5A) and pro-MMP-2 protein levels. Gelatin zymography was done to assess MMP-2 activity in cultured medium from pc.DNA3.1-sCLU transfected HepG2 cells, and we observed a significant increase in MMP-2 activity (Figure 5C).

### 2.6. Effect of *sCLU* Gene on E-Cadher in Expression

**Figure 6:**
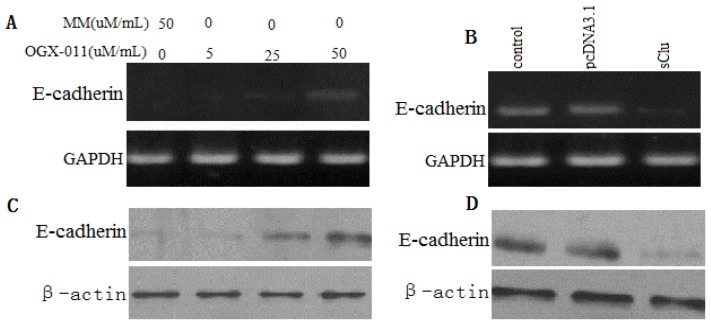
Effects of *sCLU* on *E-cadherin* mRNA and protein expression. (**A**) Semi-quantitative RT-PCR shows mRNA expression of *E-cadherin* in HCCLM3 cells treated with increasing concentrations of OGX-011. MM was used as a control in parallel. GAPDH was used as an internal control; (**B**) Semi-quantitative RT-PCR shows mRNA expression of *E-cadherin* in HepG2 cells transfected with pc.DNA3.1-sCLU for 36 h. pc.DNA3.1 was used as a control in parallel. GAPDH was used as an internal control; (**C**) Western blot analysis shows E-cadherin protein expression in HCCLM3 cells treated with increasing concentrations of OGX-011. MM was used as a control in parallel; (**D**) Western blot analysis shows E-cadherin protein expression in HepG2 cells transfected with pcDNA3.1-sCLU for 36 h. pc.DNA3.1 was used as a control in parallel. β-actin was used as an internal control.

E-cadherin is a marker of invasion and metastasis, and has been shown to be down-regulated in many malignancies, including HCC. RT-PCR was done to confirm the up-regulation of E-cadherin transcripts in *sCLU* knockdown HCCLM3 cells (Figure 6A) and the down-regulation of E-cadherin transcripts in HepG2 cells overexpressing *sCLU* (Figure 6B). We also observed that knockdown of *sCLU* in HCCLM3 cells inhibited E-cadherin protein levels (Figure 6C) and *sCLU* overexpression in HepG2 cells increased protein levels of E-cadherin (Figure 6D). These data suggest the involvement of *sCLU* in the regulation of E-cadherin.

### 2.7. Discussion

HCC invasiveness is a key step that leads to metastasis, resulting in a poor prognosis [29]. Therefore, it is of great value to study the molecular mechanism of HCC invasiveness. Recently, increasing evidence has shown that EMT, a process first identified in embryogenesis [30], mediates tumor progression, including local invasion, spreading through the circulation and metastasis.

Clusterin (CLU) is implicated in diverse cellular processes, yet its genuine molecular function remains undefined. CLU expression has been associated with various human malignancies [19,23], yet the mechanisms by which CLU promotes cancer progression and metastasis are not elucidated. It has shown that clusterin may regulate EMT and aggressive behavior of human lung adenocarcinoma cells through modulating *E-cadherin* expression [26]. However, the role of clusterin in human HCC metastasis has yet to be elucidated.

In this study, we first reported that clusterin expression in various HCC cell lines with different metastatic potentials. As shown in Figure 1, we found that clusterin was overexpressed in metastatic cell lines when compared with nonmetastatic primary ones. The above results strongly suggested the role of clusterin in HCC metastasis.

In the present study, we also observed that the clusterin gene controls the invasiveness of human HCC cells under *in vitro* conditions through the transcriptional regulation of *MMP-2* and *E-cadherin*. This report demonstrates that clusterin regulates *MMP-2* and *E-cadherin* genes in human HCC cells.

In this study, we show that overexpression of the clusterin gene increases the invasiveness of HCC cells, whereas its suppression reverses this effect. These data provide evidence that the clusterin gene may be associated with the invasion and metastatic spread of cancerous cells during progression of human HCC.

HCC cell invasion of distant sites causes metastatic disease and is the major cause of HCC-related deaths in humans [4]. Degradation of the extracellular matrix (ECM) is required by tumor cells to invade distant tissues, and recent metastatic effects of clusterin have been linked with ECM destruction in some cancer types [26]. MMPs are known to degrade the ECM by proteolysis, and a positive correlation has been shown to exist between MMP expression levels and liver metastasis [31,32]. Recently, we showed that *MMP-2* is up-regulated during the progression of HCC metastasis to the lung in mice [32]. We also previously showed that clusterin gene suppression significantly decreased the expression of *MMP-2*, and overexpression of the clusterin gene significantly increased *MMP-2* expression. Moreover, we observed that suppression of the clusterin gene caused a decrease in the proteolytic activity of MMP-2 protein, whereas overexpression of clusterin caused the reverse effect, suggesting that transcriptional activation of *MMP-2* is regulated by the clusterin gene in HCC cells.

E-cadherin is a tumor suppressor protein that is emerging as one of the caretakers of the epithelial phenotype [33]. The loss or down-regulation of *E-cadherin* has been frequently reported in metastatic and invasive carcinomas, and is hypothesized to be a critical step in the induction of an epithelial to mesenchymal transition (EMT) [34–36]. *E-cadherin* expression has been reported to be decreased in several cancer types, including hepatocellular carcinoma [37]. Clusterin protein has been reported to promote invasive growth of lung cancer cells through modulation of E-cadherin activity [20]. Here, we present data showing that the overexpression of *sCLU* is associated with decreased levels of E-cadherin. In addition, the knockdown of clusterin gene expression is associated with increased levels of *E-cadherin*.

## 3. Materials and Methods

### 3.1. Cell Culture

Human HCC cell lines with low metastatic capacity (SMCC7721 and HepG2) was from ATCC (Rockville, MD), and high metastatic capacity (HCCLM3 and MHCC97-H) was kindly gifted from professor Liang (Liver cancer research center, Zhong Shan Hospital, Shanghai, China). They were grown as a monolayer culture in Dulbecco’s modified Eagle’s medium, supplemented with 10% fetal calf serum, 100 U/mL penicillin, and 50 μg/mL streptomycin. All these cells were cultured at 37 °C in a 5% CO_2_, 95% air environment in humidified incubators.

### 3.2. Antisense Oligonucleotide

OGX-011 is a second generation 21-mer oligonucleotide with a 20-*O*-(2-methoxy)-ethyl modification, generously provided by OncoGenex Technologies (OncoGenex, Vancouver, Canada). The sequence of OGX-011 targets the first initiation site of exon II of the human clusterin gene (5′-CAGCAGCAGAGTCTTCATCAT-3′). A 20-*O*-(2-methoxy)-ethyl gapmer mismatch (MM) control oligonucleotide (5′-CAGCGCUGACAACAGUUUCAU-3′) was generously provided by ISIS Pharmaceuticals (ISIS, Carlsbad, CA). HCCLM3 cells were incubated with OGX-011 or MM (5–50 μg/mL) for 48 h.

### 3.3. pc.DNA3.1-sCLU Plasmid Construction and Transfection

Full-length human clusterin cDNA was generated by RT-PCR from normal human fibroblast total RNA using the 5′-GACTCCAGAATTGGAGGCATG-3′ and 5′-ATCTCACTCCTCCCGGTGCT-3′ primers and cloned into pGEM T-easy (Promega, Southampton, UK). From this vector, clusterin full-length cDNA was then subcloned into the pc.DNA3.1 vector (Invitrogen, Carlsbad, CA) that was previously digested with *Hin*dIII and treated with calf intestinal alkaline phosphatase to produce pc.DNA3.1-sCLU. The resulting construct was verified by direct sequencing. Empty pc.DNA3.1 was used for generating mock control clones. Both expression vectors were completely sequenced before use. For transfection studies, HepG2 cells were plated at a density of 1 × 10^6^ cells per well in six-well plates and incubated for 24 h in complete medium. The cells were then transfected with 2–4 μg of the *sCLU* construct using an Amaxa transfection kit (Gaithersburg, MD). For controls, the same amount of empty vector pc.DNA3.1 vector (as positive control for transfection) was also transfected.

### 3.4. Western Blot Analysis

Whole cells were resuspended in buffer containing 1% SDS and 1% dithiothreitol, heated to 100 °C for 5 min, fractionated by 10% SDS-PAGE, and transferred to PVDF membranes (Immobilon P, Millipore, Watford, UK). The membranes were incubated in blocking solution for 1 h, and then incubated overnight in primary antibodies against sCLU (dilution 1:100; Cell Signaling Technology, Danvers, MA), MMP-2 (dilution 1:200; Biosynthesis Biotechnology, Beijing, China), and E-cadherin (dilution 1:150; BD Transduction Laboratories, Bedford, MA). After 10 min washes in 0.1% Tween-20 in TBS (T-TBS), the membranes were incubated in a 1:5000 dilution of anti-mouse secondary antibody (Sigma, Dorset, UK) diluted in blocking solution for 60 min. The blots were then washed again 15 min in T-TBS and detected by chemiluminescence (BM chemiluminescence substrate, Boehringer, Lewes, UK). For protein staining, Ponceau S solution 0.1% (*w*/*v*) in 5% acetic acid (Sigma, Dorset, UK) was used. Densitometric measurements of bands were performed using the UN-SCAN-IT digitizing scientific software. All experiments were repeated three times with similar results.

### 3.5. Semi-Quantitative PCR

PCR reactions were carried out using forward and reverse primer combinations for *sCLU* (sense: 5′-TGCATGAAGTTCTACGCACG-3′; antisense: 5′-TTGTTGGTCGAACAGTCCAC-3′), *MMP-2* (sense: 5′-GACAGCGGTACAGTTCATGAGCA-3′, antisense: 5′-AGGTACGTCAGTCTTATCTGTC-3′), *E-cadherin* (sense: 5′-GGAAGTCAGTTCAGACTCCAGCC-3′) (antisense: 5′-AGGCCTTTTGACTGTAATCACACC-3′) and *glyceraldehyde-3-phosphate dehydrogenase* (GAPDH); sense: 5′-AATCCCATCACCATCTTCCAGGAG-3′, antisense: 5′-GCATTGCTGATGATCTTGAGGCTG-3′). PCR reaction standardization kits were obtained from Epicentre. The cDNA was amplified with an initial denaturation at 94 °C for 2 min followed by sequential cycles of denaturation at 94 °C for 45 s, annealing at 59 °C for 45 s, and extension at 72 °C for 1 min for 30 cycles, with a final extension at 72 °C for 7 min. Densitometric measurements of band generated by RT-PCR were performed using UN-SCAN-IT. All experiments were repeated three times with similar results.

### 3.6. Gelatin Zymography

An equal number of cells (1 × 10^6^) were treated with 5–50 μM/mL OGX-011 (48 h) or transfected with pcDNA3. 1-sCLU plasmid (36 h). The conditioned media were harvested, concentrated, and electrophoresed (10 μg of protein) under non-reducing conditions. The gelatinolytic activity of MMP-2 was determined by employing a zymography kit (Invitrogen) per the vendor’s protocol.

### 3.7. *In Vitro* Chemoinvasion Assay

36 h after transfection with pc.DNA3.1-sCLU plasmid, or treatment with 50 μmol/mL OGX-011 for 48 h, cells were collected, resuspended in culture medium, and incubated in a chemoinvasion chamber kit containing a polycarbonate filter coated with Matrigel (BD Biosciences) for 24 h. In the upper chamber, 30,000 cells were seeded in fetal bovine serum-free culture media and the lower chamber contained culture media containing 10% fetal bovine serum as a chemoattractant. The cells were allowed to migrate for 24 h, after which the chamber was washed with PBS and cells were visualized per the manufacturer’s instruction. To quantitate the migratory cells, the invasion chamber was dipped in 10% acetic acid, and the resultant solution was spectrophotometrically read at 540 nm according to the vendor’s protocol [27].

### 3.8. Statistical Analysis

Student’s test for independent analysis was applied to evaluate differences between transfected and non-transfected cells with respect to *in vitro* invasive properties and expression of *MMP-2* and *E-cadherin*. Statistical analyses were carried out by using SPSS, version 10.0 where *p* < 0.05 was considered significant.

## 4. Conclusion

We demonstrated that the clusterin gene controls invasion of human HCC cells, at least in part through the transcriptional regulation of *MMP-2* and *E-cadherin* genes. We suggest that the control of invasion and metastasis *in vitro* through suppression of the clusterin gene may contribute to a novel therapeutic approach against HCC. This approach could be realized through development of specific clusterin inhibitors or use of a gene therapy approach.
