# Correction: Liang, J., *et al*. Antisense Oligonucleotide Against Clusterin Regulates Human Hepatocellular Carcinoma Invasion Through Transcriptional Regulation of Matrix Metalloproteinase-2 and E-Cadherin. *Int. J. Mol. Sci.* 2012, *13*, 10594–10607

**DOI:** 10.3390/ijms14036516

**Published:** 2013-03-22

**Authors:** Dong Chen, Yan Wang, Kejun Zhang, Xuelong Jiao, Bomin Yan, Jun Liang

**Affiliations:** 1 Department of General Surgery, the Affiliated Hospital of Qingdao Medical College, Qingdao University, Qingdao 266003, China; E-Mails: chendong.sdqd@yahoo.com.cn (D.C.); wlsdermyy@163.com (K.Z.); Jiaoxuelong@163.com (X.J.); 2 Department of Endodontics, School of Stomatology, Shandong University, Jinan 250012, China; E-Mail: wangyan1965@sdu.edu.cn; 3 Department of Oncology, the Affiliated Hospital of Medical College, Qingdao University, Qingdao 266003, China; E-Mail: Yanbm818@yahoo.com.cn

The original version of the paper reports that “*OGX-011 is a second generation 21-mer oligonucleotide with a 20-O-(2-methoxy)-ethyl modification, generously provided by OncoGenex Technologies (OncoGenex, Vancouver, Canada)*” [1] (p. 10602). OGX-011 was not provided by OncoGenex Technologies directly. Therefore, we would like to correct the wording to: “OGX-011 was obtained without the benefit of an agreement with OncoGenex, or The University British Columbia, or any other party”. The authors would like to apologize for any inconvenience this may have caused to the readers of this journal.
